# Predicting Mortality in Older Adults Using Comprehensive Geriatric Assessment: A Comparative Study of Traditional Statistics and Machine Learning Approaches

**DOI:** 10.3390/diagnostics15192491

**Published:** 2025-09-30

**Authors:** Esin Avsar Kucukkurt, Esra Tokur Sonuvar, Dilek Yapar, Yasemin Demir Avcı, Irem Tanriverdi, Andisha Behzad, Pinar Soysal

**Affiliations:** 1 Department of Internal Medicine, Faculty of Medicine, Akdeniz University, Antalya 07070, Türkiye; esinavsar@akdeniz.edu.tr; 2 Medical Data Science, Institute for Medical Informatics, Statistics, and Epidemiology, Leipzig University, 04107 Leipzig, Germany; esra.tokursonuvar@medizin.uni-leipzig.de; 3 Medical Informatics, Institute of Health Science, Akdeniz University, Antalya 07070, Türkiye; dilekceliker@outlook.com; 4 Department of Public Health Nursing, Faculty of Nursing, Akdeniz University, Antalya 07070, Türkiye; ydemir@akdeniz.edu.tr; 5 Department of Geriatric Medicine, Faculty of Medicine, Bezmialem Vakif University, Istanbul 34093, Türkiye; gr.iremmtanriverdi@gmail.com; 6 Department of Internal Medicine, Faculty of Medicine, Bezmialem Vakif University, Istanbul 34093, Türkiye; andish.behzad@gmail.com

**Keywords:** machine learning, mortality prediction, geriatric population, comprehensive geriatric assessment, neural networks

## Abstract

**Objective:** The objective was to evaluate the ability of Comprehensive Geriatric Assessment (CGA) parameters to predict all-cause mortality in older adults using both traditional statistical methods and machine learning (ML) approaches. **Methods:** A total of 1.974 older adults from a university hospital outpatient clinic were included in this study. Ninety-six CGA-related variables encompassing functional and nutritional status, frailty, mobility, cognition, mood, chronic conditions, and laboratory findings were assessed. Cox proportional hazards regression and six ML algorithms (logistic regression, support vector machine, decision tree, random forest, extreme gradient boosting, and artificial neural networks) were employed to identify mortality predictors. Model performance was evaluated using area under the curve (AUC), sensitivity, and F1-score. **Results:** During a median follow-up of 617 days (interquartile range [IQR]: 297–1015), 430 participants (21.7%) died. Lower Lawton instrumental activities of daily living scores, unintentional weight loss, slower gait speed, and elevated C-reactive protein levels were consistent mortality predictors across all models. The artificial neural network demonstrated the highest predictive performance (AUC = 0.970), followed by logistic regression (AUC = 0.851). SHapley Additive explanations (SHAP) analysis confirmed the relevance of these key features. **Conclusions:** CGA parameters provide robust prognostic information regarding mortality risk in older adults. Functional decline and inflammation markers offer greater predictive power than chronological age alone in assessing overall health and survival probability.

## 1. Introduction

As populations worldwide continue to age at an unprecedented pace, accurate mortality prediction in older adults has become a cornerstone of precision geriatrics and health system planning. Older individuals often present with complex, multifactorial health conditions that are insufficiently captured by traditional disease-centric models. In response to this complexity, the Comprehensive Geriatric Assessment (CGA) has been widely adopted as a multidimensional, interdisciplinary tool that evaluates key domains of aging including medical, functional, cognitive, nutritional, and psychosocial factors. CGA reduces nursing home admission, risk of falls, and pressure sores in hospital medical setting; decreases the risk of delirium in hip fracture; decreases the risk of physical frailty and support better management of pain in community-dwelling older adults [1,2]. On the other hand, CGA or the components or CGA, have consistently demonstrated prognostic value for adverse outcomes such as institutionalization, hospitalization, and mortality [3,4]. Indeed, prevalence and co-incidence of geriatric syndromes are quite common in older patients with chronic kidney disease, cancer, and dementia [5,6,7]. Therefore, the importance of CGA is progressively increasing not only for geriatricians but also for other specialists who commonly deal with older patients, including nephrologists, neurologists, and oncologists [8,9].

Today, advanced analytical techniques such as artificial intelligence and machine learning (ML) enable more in-depth interpretation of CGA data and facilitate the generation of personalized predictions [10]. These technologies present significant opportunities to model the complex health dynamics of older adults and to enhance clinical decision support systems [10,11]. There are a limited number of studies on this subject in the literature. However, it is observed that these studies are often conducted using a restricted number of CGA parameters and within specific patient populations, such as older adults with cancer [12,13]. To address this knowledge gap, we conducted a cohort study between 2019 and 2024 involving individuals aged 65 years and older who visited a tertiary care hospital’s outpatient geriatric clinic. Unlike acutely hospitalized cohorts, this relatively stable, ambulatory population provides a unique opportunity to explore domain-specific predictors of mortality in real-world community-dwelling older adults. In this context, we applied both traditional statistical methods (e.g., Cox proportional hazards models) and supervised ML algorithms, including random forests (RFs) and gradient boosting machines to model all-cause mortality. Accordingly, we employed both a classical survival model (Cox proportional hazards) and modern ML classifiers in parallel—the former to account for time-to-event data and derive hazard ratios, and the latter to capture non-linear patterns and classification performance at a fixed time horizon—in order to provide a comprehensive assessment of mortality risk from complementary perspectives. ML techniques not only allow for the modeling of complex, non-linear relationships among variables but also facilitate domain level interpretability through explainable AI tools such as SHapley Additive exPlanations (SHAP) [14,15].

The novelty of this study lies in its dual contribution: (1) systematically comparing the performance of classical survival models and modern ML approaches in predicting mortality among ambulatory older adults, and (2) identifying the most influential CGA subdomains driving mortality risk using explainable ML. (3) The results of this study may contribute to the targeted planning of preventive health services at the community level by determining the areas affecting the mortality risk in older individuals.

The primary objective is to enhance our understanding of domain specific mortality prediction and to inform more personalized, efficient, and interpretable applications of CGA in outpatient geriatric care.

## 2. Materials and Methods

This retrospective cohort study was conducted using electronic health records from Bezmialem University Hospital, including all patients who presented to the geriatrics outpatient clinic between January 2017 and December 2024. A total of 1.974 patients aged 65 years and older were included. The mean age of the cohort was 82.14 years, and 29.13% of the patients were male. Among them, 430 (21.7%) died during follow-up. The outcome of interest was all-cause mortality, coded as a binary variable (0 = alive, 1 = deceased). Time-to-event was calculated as the number of days between the first CGA visit and the date of death or the last recorded follow-up. The median follow-up duration was 617 days (interquartile range [IQR], 297–1015 days).

### 2.1. CGA Variables

All predictor variables (CGA domains and laboratory biomarkers) [16,17,18,19,20,21,22,23] were measured at baseline, defined as the patient’s first CGA visit. Mortality status was then ascertained during follow-up, with time-to-event calculated from this baseline visit to the date of death or censoring at last contact. The dataset comprised 96 variables derived from CGA domains, including:

Demographics: age, sex, marital status, and caregiving status

Clinical status: smoking history, polypharmacy, number and type of comorbidities (e.g., coronary artery disease, diabetes mellitus, dementia).

Functional status: Lawton Instrumental Activities of Daily Living (IADL), Barthel ADL.

Gait and balance/Fall risk Assessment: Timed Up and Go (TUG) test, Tinetti balance and gait scale.

Cognitive and mood assessments: Mini Mental State Examination, Geriatric Depression Scale.

Nutritional status: Mini Nutritional Assessment scale was used for evaluation of nutritional status.

Frailty phenotype: Based on Fried’s criteria (weight loss, exhaustion, physical activity, gait speed, grip strength).

Laboratory biomarkers: C-reactive protein (CRP), albumin, hemoglobin, creatinine, and others (All variables are shown as in Supplementary Table S1).

Variables with more than 30% missing data were excluded. For the remaining variables, missing values were imputed using median imputation. As a sensitivity analysis, we also performed multiple imputations (five imputed datasets with pooled estimates), which yielded results highly consistent with the primary analyses. The outcome (mortality) was imbalanced; therefore, the Synthetic Minority Oversampling Technique (SMOTE) was applied.

### 2.2. Survival Analysis

Survival probabilities were estimated using the Kaplan–Meier method to assess differences in survival across subgroups defined by sex, smoking status, caregiver support, and age categories. A complete case analysis was applied, whereby any patient with missing data in the variables used for survival analysis was excluded. Group differences were statistically evaluated using the log-rank test, with a significance threshold of *p* < 0.05. Multivariable Cox proportional hazards regression was used to identify independent predictors of mortality. The proportional hazards assumption was tested using Schoenfeld residuals, and multicollinearity was examined using variance inflation factors (VIF). The discriminatory performance of the model was assessed using the concordance index (C-index) and the area under the ROC curve (AUC). Results were reported as hazard ratios (HR) with 95% confidence intervals (CI). Variables included in the Cox model were selected based on both clinical relevance and univariate log-rank significance.

### 2.3. Machine Learning Models

**Figure 1:**
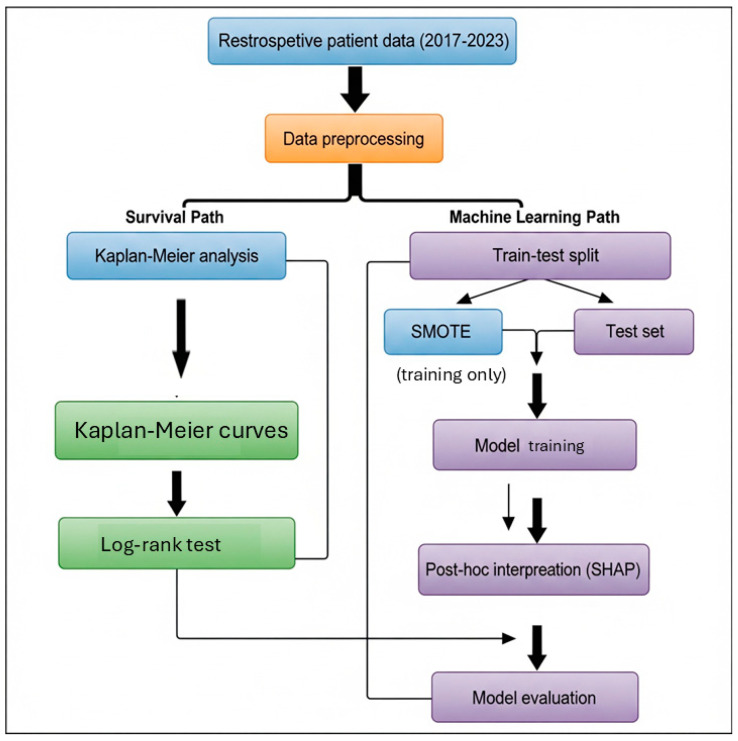
Study Flow Chart.

Following survival analysis, several supervised ML algorithms were applied to build predictive models for mortality. We selected a spectrum of ML algorithms to balance interpretability and complexity: logistic regression (LR) as a baseline linear model, decision tree (DT) and support vector machines (SVM) [24] as examples of traditional classifiers, RF and XGBoost/LightGBM [25,26] as high-performing ensemble methods, and an artificial neural networks (ANN) to capture complex non-linear patterns. This diverse selection reflects commonly used approaches in clinical prediction studies and allows comparison across model complexities. These included LR, ANN [27], RF [28], XGBoost, LightGBM, and SVM. For the ML classification, mortality was defined as death by the end of the study period; patients alive at last contact were treated as non-events (censored cases labeled as ‘survivors’) for the binary outcome. Although advanced survival ML algorithms (e.g., random survival forests, DeepSurv) are capable of directly handling censoring, our intent in this study was to directly compare Cox regression (time-to-event) with standard classification algorithms at a fixed time horizon. This design allowed us to evaluate complementary perspectives rather than to reproduce survival modeling within an ML framework. For ML analyses, missing values were handled using median imputation to preserve the dataset’s completeness. The data were randomly split into training (80%) and testing (20%) sets. To reduce the risk of overfitting, model hyperparameters were tuned via 5-fold cross-validation on the training set. Regularization was applied where applicable (e.g., penalty terms for LR and SVM, pruning parameters for DT, tree depth and learning rate constraints for ensemble models), and an early stopping criterion (based on validation loss) was implemented for the ANN to prevent over-training. All data preprocessing steps were confined to the training set to prevent information leakage. Specifically, imputation values (median/mode) were calculated from the training set only and then applied to the test set. SMOTE oversampling was restricted to the training subset after the split, and never applied to the test set. Feature importance analyses (e.g., SHAP interpretation [29]) were performed within cross-validation folds of the training data only. The held-out test set remained untouched until final model evaluation. To address class imbalance, the SMOTE was applied only on the training subset after the split for algorithms sensitive to imbalance (e.g., ANN, LR, SVM). The test set was not oversampled or otherwise altered, ensuring independence and preventing any data leakage. Numerical variables were standardized, and categorical variables were label encoded as appropriate. Feature importance was assessed using SHAP values derived from the trained tree-based models, to provide interpretability of predictors rather than to guide feature selection. All models were trained using the complete feature set (after handling missing data as described). SHAP analyses were conducted only on the training folds within cross-validation to ensure that no information from the independent test set influenced model development. Thus, SHAP did not affect model specification or training but was applied exclusively to enhance interpretability after model development. Model performance was evaluated using accuracy, sensitivity, specificity, F1-score, and AUC values obtained from ROC curve analysis. To further ensure robustness, hyperparameter tuning was conducted via 5-fold cross-validation within the training data, and we additionally report mean ± SD AUC across folds as an estimate of internal variability. For the independent test set AUC, 95% CIs were calculated using stratified bootstrap resampling with 1000 replicates. Performance metrics were compared across models to assess classification effectiveness. Feature importance distributions and ROC curves were visualized to support interpretation of the results. The overall modeling pipeline is summarized in the study flow chart (see Figure 1). Model calibration was assessed using the Brier score, a metric that measures the mean squared difference between predicted probabilities and actual outcomes. Lower scores reflect more accurate and reliable risk estimation.

All analyses were carried out using both Python (version 3.9) and R (version 4.3.1) [30] to ensure analytical reproducibility and cross-validation of results. Python packages included pandas for data handling, scikit-learn for ML implementation, lifelines for survival analysis, and matplotlib and seaborn for data visualization. In R, packages such as survival, RF, caret, and survminer were utilized for Cox regression, model training, and survival curve generation. The study protocol was approved by the Non-Interventional Research Ethics Committee of Bezmialem University (approval number: E-54022451-050.04-183065, dated 18 February 2025) and was conducted in accordance with the Declaration of Helsinki. The authors declare that there is no conflict of interest regarding the publication of this study.

## 3. Results

The study included 1.974 patients, of whom 430 (21.7%) died during follow-up. Baseline characteristics were compared between survivors and non-survivors to explore group differences. Deceased patients were older on average, more often female, and had higher rates of dementia, cardiovascular conditions (CAD, CHF), recent falls, and functional dependency (*p* < 0.001 for all). These findings are summarized in Table 1. A more detailed breakdown of all features is provided in Supplementary Table S1.

Survival analysis was conducted using both Kaplan–Meier and Cox proportional hazards (CoxPH) models to identify predictors of mortality. The median follow-up duration was calculated based on the difference between admission and death or censoring dates.

**Figure 2:**
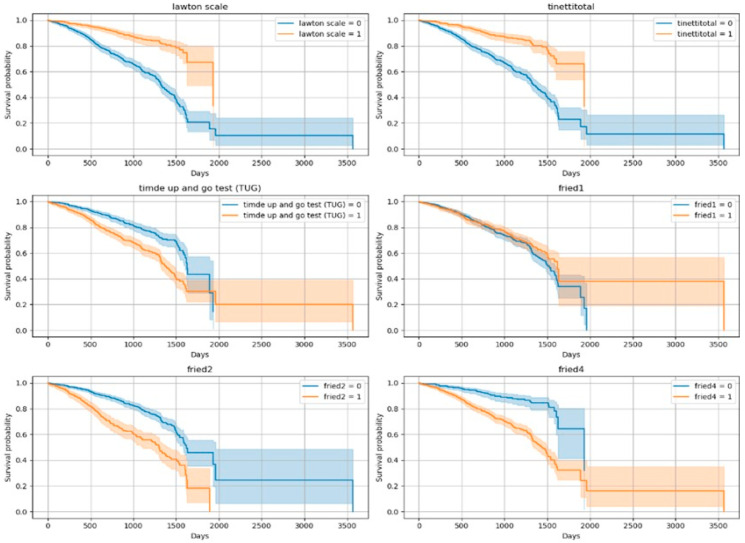
Kaplan–Meier Curves for Top 10 Variables (Log-Rank Significant)-1. (Lawton scale shows dependency of Instrumental Daily Living Activities (due to the non-normal distribution of the data, variables were categorized into two groups according to the median); Tinetti total shows gait and balance functions/Fall risk (<24/28 score is accepted increased fall risk); TUG test ≥ 13.5 s are defined as a risk of falling; fried1 criteria: self-reported exhaustion; fried2 criteria: unintentional weight loss of 4.5 kg or 5% of body weight in the prior year; fried4 criteria: walking speed < 0.8 m/s).

**Figure 3:**
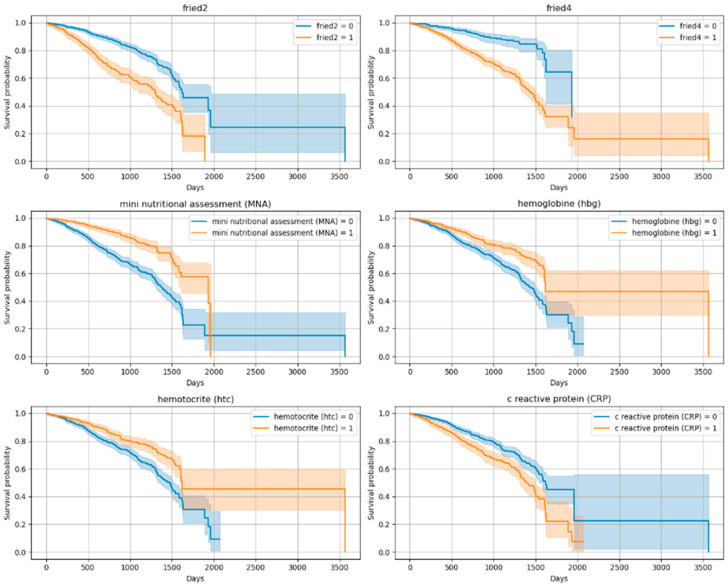
Kaplan–Meier Curves for Top 10 Variables (Log-Rank Significant)-2. (fried2 criteria: unintentional weight loss of 4.5 kg or 5% of body weight in the prior year; fried4 criteria: walking speed < 0.8 m/s; Mini Nutritional Assessment < 24/30; hemoglobin < 12.3 g/dL and hematocrit: 37.5% (According to our sample, the weighted average cut-off value was determined), CRP (variables were categorized into two groups according to the median).

The Kaplan–Meier curves revealed significant differences in survival across functional and clinical subgroups. Variables showing the most prominent separation included the Lawton IADL scale, Tinetti balance and gait test, TUG test, and frailty criteria (fried1: self-reported exhaustion; fried2: unintentional weight loss of 4.5 kg or 5% of body weight in the prior year; fried4: walking speed < 0.8 m/s). Lower functional scores and positive frailty status were associated with significantly reduced survival times (*p* < 0.001 for all). In addition to these, malnutrition risk (MNA), inflammatory markers (CRP, Hb, Ht), and cardiovascular conditions (CAD, CHF) also emerged as strong mortality predictors. Figure 2 and Figure 3 show the Kaplan–Meier curves for the top predictors.

**Figure 4:**
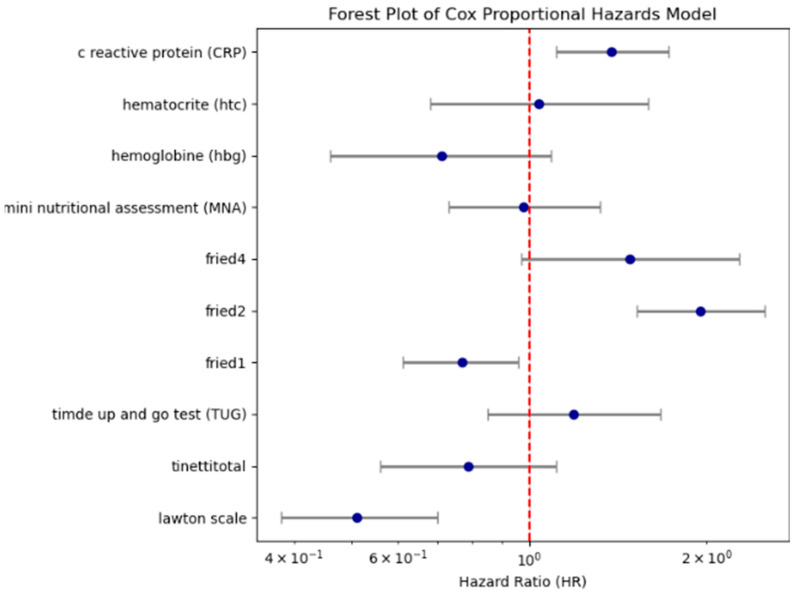
Forest plot showing hazard ratios and 95% confidence intervals for key Cox regression predictors. (Lawton scale shows dependency of Instrumental Daily Living Activities (due to the non-normal distribution of the data, variables were categorized into two groups according to the median); fried2 criteria: unintentional weight loss of 4.5 kg or 5% of body weight in the prior year; fried4 criteria: walking speed < 0.8 m/s; CRP (variables were categorized into two groups according to the median; fried1: self-reported exhaustion; Tinetti total shows gait and balance functions/Fall risk (<24/28 score is accepted increased fall risk); TUG test ≥ 13.5 s are defined as a risk of falling; Mini Nutritional Assessment < 24/30; hemoglobin < 12.3 g/dL and hematocrit: 37.5% (According to our sample, the weighted average cut-off value was determined).

Following this, a multivariable Cox regression was performed using clinically relevant and log-rank–significant variables. Reduced functional independence (Lawton scale: HR = 0.51), frailty (fried2: HR = 1.95), and systemic inflammation (CRP: HR = 1.38) emerged as independent predictors of mortality. The model achieved a C-index of 0.71. These results are shown in Table 2 and Figure 4, with permutation-based variable importance illustrated in Supplementary Figure S1.

To enhance predictive performance, multiple supervised ML algorithms were employed under two distinct modeling strategies: (1) the full dataset with imputed missing values and class balancing via SMOTE, and (2) a complete-case subset comprising 28 SHAP-selected features.

**Figure 5:**
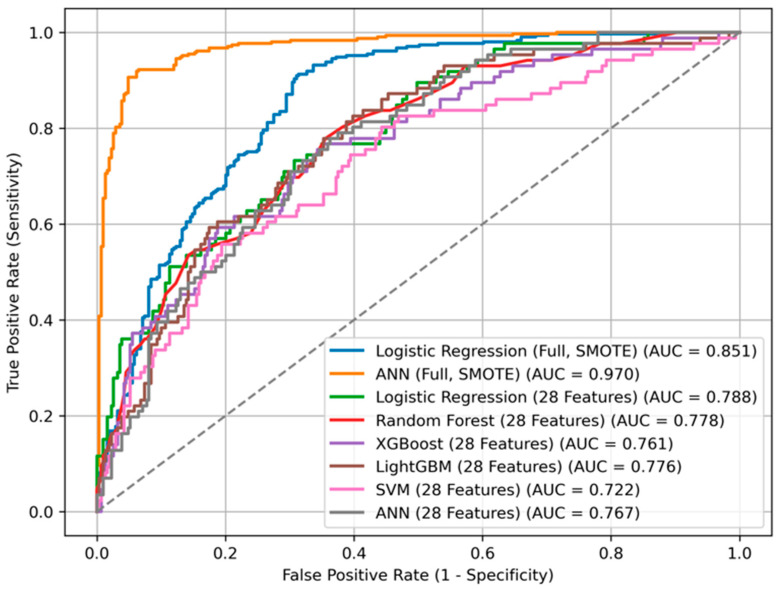
ROC curves for different machine learning models predicting mortality.

The ANN trained on the imputed and SMOTE-balanced dataset yielded the highest overall performance (AUC = 0.970), with high accuracy (87.2%), sensitivity (88.3%), and F1-score (0.874). LR also showed strong discriminative ability (AUC = 0.851). Detailed performance metrics for all models are provided in Table 3 and illustrated in Figure 5. Cross-validation results confirmed the stability of these estimates; for example, ANN achieved a cross-validated AUC of 0.962 ± 0.012 across training folds. For the independent test set, 95% CIs were calculated using 1000 stratified bootstrap replicates (e.g., ANN test AUC 0.970, 95% CI 0.958–0.981; LR 0.851, 95% CI 0.832–0.868). These complementary evaluations support the robustness of our performance estimates and provide a measure of internal variability and uncertainty. In addition to discrimination performance, model calibration was assessed using the Brier score, which quantifies the accuracy of probabilistic predictions. The ANN achieved the lowest Brier score (0.079), followed by RF (0.104) and LR (0.148), indicating that the ANN’s predicted probabilities were more closely aligned with actual outcomes. These results suggest that the best-performing models in terms of discrimination also demonstrated reasonably good calibration in this dataset (Table 3). Among the tested models, the RF algorithm yielded the best calibration (Brier score = 0.159), followed by XGBoost (0.165) and LR (0.174). The artificial neural network demonstrated comparatively poorer calibration (0.182), despite its high discrimination. These findings suggest that tree-based models provided more reliable probability estimates of mortality compared to neural networks. We further assessed clinical utility using decision curve analysis (DCA). Across clinically plausible threshold probabilities (0.10–0.30), ANN, LR, and RF all demonstrated modest positive net benefit compared to ‘treat-none’ and clearly outperformed the ‘treat-all’ strategy, which was consistently harmful in this cohort (Supplementary Figure S2). Net benefit values were similar across models, suggesting comparable clinical usefulness despite differences in AUC.

Sensitivity analyses using multiple imputation (MI) confirmed the robustness of our findings. As shown in Table 3, model performance metrics under MI were nearly identical to those obtained with the primary analyses (median imputation for ML and complete-case analysis for Cox). For example, the ANN achieved an AUC of 0.964 under MI compared to 0.970 with median imputation, and LR yielded an AUC of 0.848 under MI compared to 0.851 with median imputation. Similarly, among the selected 28-variable models, performance differences between MI and complete-case analyses were negligible (<0.01 in AUC across models). These results indicate that our conclusions are robust to the method of handling missing data.

In contrast, the complete-case analysis resulted in an overall reduction in predictive performance. LR maintained the highest performance (AUC = 0.788), followed closely by RF (AUC = 0.778), LightGBM (AUC = 0.776), and ANN (AUC = 0.767). SVM exhibited notably poor results in this setting, achieving an AUC of only 0.722 and a sensitivity of merely 3.5%.

Feature importance derived from SHAP values in tree-based models (particularly XGBoost) identified frailty, systemic inflammation (e.g., CRP), and functional scores (e.g., Lawton, Tinetti) as the most influential predictors. These findings are in line with conventional statistical approaches, including Cox regression and Kaplan–Meier analysis. The convergence between model-driven feature selection and clinical plausibility enhances the interpretability and reliability of the results (Figure 5).

## 4. Discussion

This study integrated classical survival analysis and modern ML techniques to identify predictors of all-cause mortality among ambulatory older adults. Across both modeling frameworks, reduced functional capacity, frailty indicators, and systemic inflammation consistently emerged as the most robust and clinically relevant risk factors underscoring the multidimensional nature of vulnerability in geriatric populations. Specifically, the IADL scale and select Fried frailty phenotype components (weight loss and reduced walking speed) were identified as the strongest independent predictors of mortality in survival analysis, reinforcing prior evidence linking frailty and functional decline to biological aging and mortality risk [17,31].

The predictive strength of these tools is particularly noteworthy given their widespread use in routine geriatric assessments. Their consistent association with mortality across both statistical and ML models highlights their potential role in early risk stratification, triage, and individualized care planning. Furthermore, systemic inflammation measured by elevated CRP levels also demonstrated a strong association with mortality. This finding supports the growing body of evidence implicating chronic low-grade inflammation (“inflammaging”) as a biological amplifier of frailty, multimorbidity, and poor late-life outcomes [32,33]. In our study, we found that elevated CRP levels and components of frailty were associated with mortality. This finding supports previous studies demonstrating the relationship between frailty and inflammatory markers [34]. Firstly, frail older people often present with coexisting factors such as disabilities and chronic medical conditions, which may contribute to elevated levels of inflammatory markers. Additionally, frail older patients typically exhibit a marked decline in innate immunity, T-cell function, and antibody production, alongside heightened mitochondrial activity and oxidative stress, which collectively contribute to elevated systemic inflammation [35]. These factors may help explain why individuals with elevated CRP levels tend to have higher mortality rates. Consistent with prior reports, we found that functional impairment and inflammation markers (e.g., IADL dependency, slow gait speed, high CRP) are among the strongest predictors of mortality [3,4]. Our study extends these findings by demonstrating their predictive power in a general ambulatory geriatric cohort using both Cox and ML methods.

Another important finding of this study is the limited prognostic value of chronological age when compared to domain-specific measures of biological vulnerability. Although age is often assumed to be a primary determinant of mortality, our models revealed that chronological age did not independently contribute to mortality risk once variables related to frailty, functional decline (reduced Lawton Instrumental activities of daily living, reduced walking speed), and inflammation were considered. This observation aligns with a growing body of literature suggesting that biological age as reflected by markers such as mobility limitations, sarcopenia, and systemic inflammation offers a more accurate reflection of physiological reserve and mortality risk than chronological age alone [33,36,37,38,39]. For instance, Levine’s phenotypic age framework and subsequent ML–derived biological age clocks have consistently outperformed chronological age in predicting morbidity and mortality across diverse populations [36,39]. Similarly, Kennedy, Berger, Brunet, Campisi, Cuervo, Epel, Franceschi, Lithgow, Morimoto, Pessin, Rando, Richardson, Schadt, Wyss-Coray and Sierra [38] argue that functional decline and cumulative damage, rather than the passage of time per se, are the hallmarks of aging. Our results reinforce these perspectives by showing that even in an ambulatory older cohort, domain-specific variables from the CGA including frailty (reduced walking speed, function (dependency on Lawton IADL), and CRP substantially outweighed age in predictive relevance. These findings support a paradigm shift in geriatric risk assessment away from age-based heuristics and toward multidimensional, clinically interpretable indicators of biological vulnerability.

One of the important results is that weight loss and malnutrition was related with mortality. Disruption in the balance between energy intake and expenditure may contribute to the development of sarcopenia and muscle weakness, conditions that are linked to decreased muscle function, slower physical performance, fatigue, frailty, and limitations in daily activities among older adults [3,40]. Therefore, nutritional screening plays a critical role in the management of older adults, enabling early detection of malnutrition and timely implementation of appropriate interventions. In practice, these results suggest that a geriatric clinic could implement a simple prognostic tool—for instance, flagging patients with poor IADL scores, weight loss, and elevated CRP for comprehensive care planning or closer follow-up. The integration of ML predictions with CGA data in electronic health records could further personalize risk assessments, though prospective evaluation of such an approach is needed. From a clinical perspective, thresholds could be set based on PPV/NPV to flag high-risk patients for closer follow-up or intervention. For instance, patients flagged by low IADL scores or high CRP could be targeted for preventive strategies such as nutritional support or exercise programs. Such threshold-based applications, however, require further validation before clinical use. Consistent with this perspective, DCA indicated that ANN, LR, and RF all provided modest positive net benefit across clinically relevant thresholds (0.10–0.30), while the ‘treat-all’ strategy was consistently harmful. This suggests that the models may offer added clinical value in guiding risk-based interventions, although external validation is required before routine use.

Machine learning models, particularly ANNs trained on imputed and SMOTE-balanced datasets, demonstrated superior discriminative performance (AUC = 0.970) compared to classical statistical models. These results align with recent literature showing the advantages of deep learning in complex clinical prediction tasks [41]. However, this high performance raises concerns about potential overfitting particularly when synthetic oversampling techniques such as SMOTE are employed. Although SMOTE is widely adopted for addressing class imbalance, it may alter the natural heterogeneity of clinical data. Therefore, as emphasized in previous studies, careful calibration and external validation are essential before deploying ML models in clinical settings [42,43].

An important strength of this study lies in the convergence of findings across methodological approaches. The top features identified by SHAP in ML models frailty components, functional status (Lawton), and inflammation (CRP) mirrored those identified in Cox regression models. This consistency supports the robustness and generalizability of these predictors and highlights the complementary value of combining domain expertise with data-driven algorithms to develop interpretable, clinically useful decision-support tools [44]. Nevertheless, this study has several limitations. Unlike Cox regression, the ML models did not explicitly account for censoring; censored individuals were treated as survivors at the end of follow-up. This simplification may introduce bias if follow-up durations vary substantially. Although survival-specific ML methods (e.g., random survival forests, neural-network–based Cox models) could address censoring, our study prioritized a direct comparison of Cox survival analysis with conventional classification ML approaches. Future research should extend our findings by applying survival ML methods to better integrate censoring into predictive modeling. In addition, while we used different strategies for handling missing data (median imputation for ML vs. complete-case analysis for Cox), the overall proportion of missing values was low (<10%). Importantly, sensitivity analyses using multiple imputation produced results nearly identical to the primary analyses, confirming that our findings are robust to alternative missing data strategies. Given that the overall missingness per variable was <10%, median imputation was considered sufficient and was retained for the final models. First, it was conducted at a single tertiary-care center, which may limit the external validity and generalizability of the findings. In addition, the models were not validated on an independent external cohort. The high performance observed, particularly the ANN (AUC = 0.97), is based solely on internal validation and may not generalize to other populations or healthcare settings. This exceptionally high ANN performance should therefore be interpreted with caution. Despite the use of cross-validation, regularization, and early stopping to reduce overfitting, such results may partly reflect dataset-specific patterns and could be overly optimistic without external validation. Accordingly, these findings should be regarded as exploratory until validated in independent cohorts. Future studies should include external validation in independent cohorts to confirm robustness and clinical utility. Moreover, while cross-validation and bootstrap resampling confirmed the stability of model estimates, we did not perform formal statistical comparisons between algorithms; therefore, reported differences should be considered descriptive rather than inferential. Second, while SMOTE improved class balance, it may introduce artificial patterns that do not fully reflect real-world patient variability. Third, although model discrimination was rigorously evaluated using metrics such as AUC and F1-score, model calibration and clinical utility (e.g., net benefit via decision curve analysis) were not formally assessed as an important direction for future research. In addition, although common comorbidities such as coronary artery disease, dementia, and diabetes were included, they did not emerge as dominant predictors. This likely reflects that in very old and multimorbid populations, geriatric domains such as frailty, functional status, and systemic inflammation capture mortality risk more strongly than single disease diagnoses. We also acknowledge that comorbidities in our dataset were recorded as binary indicators without severity indices, which may have limited their predictive contribution. Future studies should integrate disease-specific severity measures alongside CGA domains to further refine risk prediction. Nevertheless, we did evaluate calibration internally using Brier scores, which showed that the ANN achieved the lowest score (0.079), followed by RF (0.104) and LR (0.148). These findings suggest that, in addition to discrimination, some of our models also provided reasonably good probability calibration in this dataset. However, external validation will be essential to confirm that these calibration properties hold in independent populations. Calibration is particularly critical for clinical applicability, as even models with excellent discrimination may fail in practice if their predicted probabilities are poorly aligned with observed risks. To enhance the applicability and robustness of these findings, future studies should incorporate multi-institutional datasets and perform prospective external validation. Further work is also needed to explore the integration of ML derived risk scores into real-time clinical workflows and evaluate their impact on clinical outcomes. Additionally, exploring the incorporation of dynamic or longitudinal CGA data might further enhance predictive accuracy and clinical utility.

## 5. Conclusions

This study highlights the value of combining traditional survival analysis with modern ML to improve mortality risk prediction in a diverse outpatient population. Functional decline, frailty components including weight loss and reduced walking speed, and systemic inflammation consistently emerged as the most influential predictors across both statistical and algorithmic approaches. Among the ML models, ANNs trained with SMOTE preprocessing demonstrated the highest discriminative performance (AUC = 0.970), clearly outperforming classical methods. Despite their complexity, these models identified similar key predictors as traditional approaches, such as the Lawton functional scale, frailty components, and CRP. This consistency reinforces the robustness of these variables and demonstrates the complementarity between data-driven and clinically informed models. LR also achieved strong performance, supporting its use in transparent and interpretable clinical applications. These findings underscore the potential of leveraging routinely collected clinical variables especially those reflecting frailty, function, and inflammation to enable early identification of high-risk patients. Future research should prioritize external validation, model calibration, and the development of clinically integrated decision-support tools that translate predictive insights into actionable care. By bridging statistical rigor with ML power, this study offers a scalable and interpretable framework for personalized mortality risk stratification in aging and comorbid patient populations.

## Figures and Tables

**Table 1:** Comparison of Demographic and Clinical Characteristics by Mortality Status in Older Adults (*n* = 1.974).

	Mortality Status (Status)		
Variables	0 N = 1544 *	1 N = 430 *	*p*-value **
Gender, Female (%)	1.139 (74%)	260 (60%)	<0.001
Age, years	81.01 (7.71)	86.19 (7.17)	<0.001
Marital Status			<0.001
Single	77 (5.0%)	44 (10%)	
Married	740 (48%)	182 (42%)	
Wife/Husband ex	699 (45%)	200 (47%)	
Widowed	28 (1.8%)	4 (0.9%)	
Caregiving Status	131 (8.5%)	12 (2.8%)	<0.001
Driving			<0.001
Never drove	1213 (79%)	330 (77%)	
Driver in the past	222 (14%)	88 (20%)	
Active driver	109 (7.1%)	12 (2.8%)	
Smoking			0.6
No smoking history	1023 (66%)	254 (59%)	
Smoker in the past	400 (26.2%)	141 (33%)	
Active smoker	116 (7.5%)	34 (7.9%)	
Number of Drugs Used	6.39 (3.50)	6.91 (3.68)	0.006
Dementia	455 (29%)	186 (43%)	<0.001
Coronary Artery disease (CAD)	252 (16%)	135 (31%)	<0.001
Congestive Heart failure (CHF)	148 (9.6%)	76 (18%)	<0.001
Benign Prostate Hyperplasia (BPH)	105 (6.8%)	52 (12%)	<0.001
Osteoarthritis (OA)	273 (18%)	45 (10%)	<0.001
Fall-1 year	624 (40%)	227 (53%)	<0.001
Dizziness	690 (45%)	177 (41%)	<0.001
Number of Nocturia	2.17 (1.96)	2.51 (2.36)	<0.001
Constipation	667 (43%)	202 (47%)	0.002
Hypertension	1081 (70%)	287 (67%)	0.4
Chronic Obstructive Lung Disease (COPD)	96 (6.2%)	41 (9.5%)	0.017
Cerebrovascular disease (CVD)	166 (11%)	65 (15%)	0.013
Parkinson Disease	128 (8.3%)	53 (12%)	0.010
Incontinence	869 (56%)	253 (59%)	0.2
Nocturia	1239 (80%)	32 (77%)	0.3
Pain	949 (61%)	214 (50%)	0.2

* n/N (%); Mean (SD) ^2^ Pearson’s Chi-squared test; ** Wilcoxon rank sum test; Fisher’s exact test.

**Table 2:** Summary—CoxPH Results.

Variable → HR	(exp(coef))	*p*-Value	Interpretation
Lawton scale	0.51	<0.005	Low functional status → Approximately 2 times higher risk of mortality
fried2	1.95	<0.005	Frailty component → 95% increased risk
fried4	1.48	0.07	Marginally significant
CRP	1.38	<0.005	High CRP → 38% increased risk
fried1	0.77	0.02	Appears to have a slight protective effect
Tinetti total	0.79	0.17	Not statistically significant
TUG	1.19	0.31	Not significant
MNA	0.98	0.89	Not significant
Hb	0.71	0.38	Not significant
Htc	1.04	0.86	Not significant

Lawton scale shows dependency of Instrumental Daily Living Activities (due to the non-normal distribution of the data, variables were categorized into two groups according to the median); fried2 criteria: unintentional weight loss of 4.5 kg or 5% of body weight in the prior year; fried4 criteria: walking speed < 0.8 m/s; CRP variables were categorized into two groups according to the median; fried1: self-reported exhaustion; Tinetti total shows gait and balance functions/Fall risk (<24/28 score is accepted increased fall risk); TUG test ≥ 13.5 s are defined as a risk of falling; Mini Nutritional Assessment < 24/30; hemoglobin < 12.3 g/dL and hematocrit: 37.5% (According to our sample, the weighted average cut-off value was determined).

**Table 3:** Comparison of machine learning model performance.

Variables Count	Missing Value Handling	Model	Accu.	Specif.	Sensitiv.	F1 Score	PPV *	NPV *	Balanced Acc.	ROC AUC	AUC (CV, #break# Mean ± SD)	AUC (95% CI)	Brier Score
ALL Features	Imputation (Median) + SMOTE	ANN	0.872	0.861	0.883	0.874	0.864	0.881	0.872	0.970	0.962 ± 0.012	(0.958–0.981)	0.079
ALL Features	Multiple Imputation (MI) + SMOTE	ANN	0.868	0.857	0.879	0.870	0.860	0.877	0.868	0.964	0.958 ± 0.013	(0.952–0.976)	0.083
All Features	Imputation (Median) + SMOTE	LR	0.841	0.826	0.847	0.826	0.834	0.853	0.838	0.851	0.846 ± 0.015	(0.832–0.868)	0.148
ALL Features	Multiple Imputation (MI) + SMOTE	LR	0.839	0.824	0.844	0.824	0.833	0.852	0.837	0.848	0.843 ± 0.016	(0.829–0.866)	0.152
Selected 28 Features	Complete Case Anal.	LR	0.813	0.974	0.233	0.351	0.714	0.823	0.603	0.788	0.787 ± 0.019	(0.770–0.809)	0.172
Selected 28 Features	MI	LR	0.815	0.971	0.237	0.355	0.718	0.826	0.606	0.790	0.788 ± 0.018	(0.771–0.810)	0.170
Selected 28 Features	Complete Case Anal.	ANN	0.808	0.951	0.291	0.397	0.625	0.828	0.621	0.767	0.765 ± 0.022	(0.746–0.792)	0.180
Selected 28 Features	MI	ANN	0.811	0.948	0.296	0.302	0.629	0.831	0.622	0.769	0.767 ± 0.020	(0.748–0.791)	0.182
Selected 28 Features	Complete Case Anal.	RF	0.800	0.977	0.163	0.262	0.667	0.807	0.570	0.778	0.775 ± 0.018	(0.760–0.800)	0.161
Selected 28 Features	MI	RF	0.802	0.975	0.169	0.268	0.670	0.810	0.573	0.780	0.778 ± 0.014	(0.762–0.802)	0.159
Selected 28 Features	Complete Case Anal.	XGBoost	0.815	0.945	0.349	0.451	0.638	0.839	0.647	0.761	0.760 ± 0.020	0.741–0.785)	0.168
Selected 28 Features	MI	XGBoost	0.817	0.943	0.353	0.455	0.642	0.841	0.658	0.763	0.761 ± 0.013	(0.744–0.786)	0.165
Selected 28 Features	Complete Case Anal.	LightGBM	0.777	0.929	0.233	0.312	0.476	0.813	0.581	0.776	0.775 ± 0.018	(0.756–0.797	0.170
Selected 28 Features	MI	LightGBM	0.779	0.927	0.238	0.317	0.480	0.816	0.582	0.777	0.776 ± 0.018	(0.757–0.798)	0.168
Selected 28 Features	Complete Case Anal.	SVM	0.782	0.990	0.035	0.065	0.500	0.787	0.513	0.722	0.720 ± 0.021	(0.700–0.747)	0.190
Selected 28 Features	MI	SVM	0.784	0.987	0.040	0.070	0.503	0.790	0.515	0.724	0.724 ± 0.021	(0.703–0.749)	0.188

* Comparison of machine learning models in predicting mortality outcomes. PPV: Positive Predictive Value, NPV: Negative Predictive Value.

## Data Availability

The data supporting the findings of this study are available from the corresponding author (P.S.) upon request.

## Supplementary Materials

The following supporting information can be downloaded at https://www.mdpi.com/article/10.3390/diagnostics15192491/s1: Table S1: Descriptive Statistics of All Features; Figure S1: Permutation Importance; Figure S2: Decision Curve Analysis.
