# Supplementary material for: Predicting Mortality in Older Adults Using Comprehensive Geriatric Assessment: A Comparative Study of Traditional Statistics and Machine Learning Approaches
Source: Diagnostics (Basel). 2025 Sep 30;15(19):2491. doi: 10.3390/diagnostics15192491 (PMC12523355; doi:10.3390/diagnostics15192491)

## Appendix

*Appendix S.1*

**Table S1:** Descriptive Statistics of All Features

| | **Mortalite Status (Status)** | | |
|---|---|---|---|
| Variables | 0 N = 1,544[1] | 1 N = 430[1] | p-value[2] |
| Gender, Female | 1,139 (74%) | 260 (60%) | <0.001 |
| Age | 81.01 (7.71) | 86.19 (7.17) | <0.001 |
| Marital Status | | | <0.001 |
| • Single | 77 (5.0%) | 44 (10%) | |
| • Married | 740 (48%) | 182 (42%) | |
| • Wife/Husband ex | 699 (45%) | 200 (47%) | |
| • Widowed | 28 (1.8%) | 4 (0.9%) | |
| Living Status | | | |
| Alone | 217 (14%) | 36 (8.4%) | |
| With wife/husband | 713 (46%) | 171 (40%) | |
| With children | 520 (34%) | 189 (44%) | |
| With formal caregiver | 72 (4.7%) | 31 (7.2%) | |
| With others (relatives, friends) | 22 (1.5%) | 3 (0.6%) | |
| Caregiving Status | 131 (8.5%) | 12 (2.8%) | <0.001 |
| Driving | | | <0.001 |
| Never drived | 1,213 (79%) | 330 (77%) | |
| Driver in the past | 222 (14%) | 88 (20%) | |
| Acitve driver | 109 (7.1%) | 12 (2.8%) | |
| Number of Drugs Used | 6.39 (3.50) | 6.91 (3.68) | 0.006 |
| Dementia | 455 (29%) | 186 (43%) | <0.001 |
| Systolic Blood Pressure, mm/Hg | 140.92 (23.51) | 137.01 (25.49) | 0.004 |
| Diastolic Hypertension Blood Pressure, mm/Hg | 76.59 (14.17) | 75.22 (13.76) | 0.061 |
| Coronary Artery Disease (CAH) | 252 (16%) | 135 (31%) | <0.001 |
| Congestive Heart Failure (CHF) | 148 (9.6%) | 76 (18%) | <0.001 |

| | Mortalite Status (Status) | | |
|---|---|---|---|
| Variables | 0 N = 1,544[1] | 1 N = 430[1] | p-value[2] |
| Benign Prostate Hyperplasia (BPH) | 105 (6.8%) | 52 (12%) | <0.001 |
| Osteoarthritis (OA) | 273 (18%) | 45 (10%) | <0.001 |
| Fall, in last 1 year | 624 (40%) | 227 (53%) | <0.001 |
| Number of Falls | 1.09 (2.27) | 1.77 (3.04) | <0.001 |
| Dizziness | 690 (45%) | 177 (41%) | <0.001 |
| Number of Nocturia | 2.17 (1.96) | 2.51 (2.36 | <0.001 |
| Diaper Need | 445 (29%) | 158 (37%) | 0.052 |
| Constipation | 667 (43%) | 202 (47%) | 0.002 |
| Lawton Instreumental ADL | 13.08 (7.80) | 6.96 (6.99) | <0.001 |
| Mini Nutritional Assessment (MNA) | 21.87 (4.92) | 18.01 (6.05) | <0.001 |
| Body Mass Index (BMA) | | | <0.001 |
| < 23 kg/m$^2$ | 125 (8.5 %) | 79 (20.9%) | <0.001 |
| ≥ 23 kg/m$^2$ | 1,339 (91%) | 300 (79%) | |
| Selfness_Health | | | |
| not as good | 428 (28%) | 119 (30%) | <0.001 |
| does not know | 156 (10%) | 98 (25%) | |
| As good | 740 (49%) | 156 (39%) | |
| Better | 185 (12%) | 25 (6.3%) | |
| Weight Loss | | | |
| More than 3 kg | 323 (21%) | 168 (41%) | <0.001 |
| ≤ 3 kg | 1188 (79%) | 240 (59%) | |
| Upper Arm Circumference, cm | 28.98 (5.16) | 26.73 (4.15) | <0.001 |
| Calf Circumference, cm | 35.90 (4.33) | 34.00 (4.78) | <0.001 |
| Height, cm | 156.76 (10.08) | 157.08 (9.87) | <0.001 |
| Weight, kg | 71.98 (14.53) | 67.78 (15.05) | 0.3 |
| Postural_Instability | | | <0.001 |
| No | 377 (25%) | 190 (49%) | <0.001 |

| | Mortalite Status (Status) | | |
|---|---|---|---|
| Variables | 0 N = 1,544[1] | 1 N = 430[1] | p-value[2] |
| Some | 170 (11%) | 50 (13%) | |
| Yes | 933 (64%) | 147 (38%) | |
| Romberg_Test | | | |
| 0 | 253 (17%) | 141 (36%) | <0.001 |
| 1 | 1,225 (83%) | 248 (64%) | |
| Tinetti Total Score | 21.86 (8.18) | 15.15 (10.21) | |
| Timed Up and Go test | 19.81 (16.15) | 35.37 (30.31) | <0.001 |
| Muscle Strength | 18.10 (10.50) | 13.85 (8.13) | <0.001 |
| Geriatric Depression Scale | 5.06 (4.08) | 5.86 (4.47) | <0.001 |
| Mini Mental State Examination | 22.33 (6.21) | 18.71 (8.15) | 0.006 |
| Education | 5.38 (4.77) | 4.65 (4.62) | <0.001 |
| EAT-10 Scale | 2.08 (4.63) | 4.48 (6.89) | 0.005 |
| Insomnia Severity Index | 11.20 (9.39) | 14.25 (10.30) | <0.001 |
| Epworth Sleepiness Scale | 5.51 (5.46) | 8.65 (6.66) | <0.001 |
| Fried Frailty Components | | | |
| Fired1:Self-reported exhaustion | 679 (48%) | 168 (53%) | <0.001 |
| Fried2:Unintentional weight loss | 323 (22%) | 170 (44%) | |
| Fried3: Reduced muscle strength | 830 (55%) | 327 (83%) | <0.001 |
| Fried4: Decreased walking speed | 848 (58%) | 314 (85%) | <0.001 |
| Fried5: Low physical activity | 974 (66%) | 358 (88%) | <0.001 |
| The Council on Nutrition Appetite Questionnaire score | 27.37 (6.21) | 24.00 (7.50) | <0.001 |
| Caregiver Gender, Female (%) | 1.091 (71%) | 369 (86%) | <0.001 |
| Caregiver age, years | 52.39 (12.88) | 53.02 (12.94) | 0,4 |
| Neuropsychiatric Inventory score | 22.45 (20.79) | 32.63 (17.84) | 0.3 |
| Laboratory Assesment | | | |
| Glucose, mg/dL | 121.70 (47.71) | 131.33 (61.20) | <0.001 |
| Urea, mg/dL | 45.14 (21.84) | 60.36 (34.26) | 0.031 |

| | Mortalite Status (Status) | | |
|---|---|---|---|
| Variables | 0 N = 1,544[1] | 1 N = 430[1] | p-value[2] |
| Creatinine, mg/dL | 1.04 (0.49) | 1.29 (0.78) | <0.001 |
| Glomeruler Filtration Rate, mL/dk/1.73m2 | 61.87 (18.90) | 54.31 (21.16) | <0.001 |
| Uric Acid, mg/dL | 5.58 (1.67) | 6.35 (2.21) | <0.001 |
| Albumin, g/L | 4.39 (2.57) | 4.39 (5.32) | <0.001 |
| Triglyceride, mg/dL | 143.65 (80.05) | 136.96 (116.48) | <0.001 |
| High Density Lipoprotein (HDL), mg/dL | 53.47 (14.22) | 48.74 (14.01) | 0.049 |
| Low Density Lipoprotein (LDL), mg/dL | 132.22 (39.40) | 119.95 (40.53) | <0.001 |
| Aspartat Transaminase (AST), U/L | 20.20 (12.03) | 24.34 (27.23) | <0.001 |
| Alanine Transaminase (ALT), U/L | 18.49 (17.75) | 19.88 (32.27) | 0.7 |
| Calcium, mg/dL | 9.55 (3.81) | 9.18 (0.66) | <0.001 |
| Magnesium, mg/dL | 1.90 (0.32) | 1.90 (0.29) | <0.001 |
| Phosphorus, mg/dL | 3.46 (0.61) | 3.38 (0.69) | >0.9 |
| Sodium, mmol/L | 139.48 (3.38) | 138.91 (3.83) | 0.004 |
| Potassium, mmol/L | 4.43 (0.50) | 4.43 (0.67) | 0.003 |
| Iron, mg/dL | 69.57 (33.89) | 51.45 (25.47) | 0.6 |
| Total Iron Binding Capacity (TIBC), ug/dL | 243.15 (137.66) | 213.09 (78.99) | <0.001 |
| White Blood Cell (WBC), thousand/mm3 | 7.75 (4.61) | 8.15 (2.73) | <0.001 |
| Hemoglobine (Hbg), g/dL | 12.62 (3.10) | 11.65 (1.89) | <0.001 |
| Hemotocrite (Htc), % | 38.91 (4.72) | 36.64 (5.41) | <0.001 |
| Mid Corpuscular Volume(MCV), fL | 88.08 (6.21) | 88.02 (6.72) | <0.001 |
| Platelet, thousand/mm3 | 251.35 (123.02) | 246.72 (92.12) | 0.6 |
| Mean Platelet Volume (MPV), fL | 8.57 (1.82) | 7.92 (1.70) | 0.11 |
| Red Cell Distribution Width RDW), % | 13.38 (1.70) | 13.97 (2.19) | <0.001 |
| C Reactive Protein (CRP), mg/dL | 10.01 (22.73) | 19.36 (32.13) | <0.001 |
| Vitamin D, ng/mL | 24.96 (15.01) | 22.91 (16.03) | <0.001 |

| | Mortalite Status (Status) | | |
|---|---|---|---|
| Variables | 0 N = 1,544[1] | 1 N = 430[1] | p-value[2] |
| Thyroxine (T4), ng/dL | 7.73 (6.58) | 7.40 (6.42) | 0.003 |
| Thyroid Stimulating Hormone (TSH), mIU/L | 2.04 (4.98) | 1.97 (2.32) | 0.3 |
| Ferritine, ug/L | 91.37 (141.82) | 150.11 (220.13) | >0.9 |
| Folate, ng/mL | 7.74 (3.98) | 7.03 (4.17) | <0.001 |
| Vitamine B12, ng/L | 492.52 (357.69) | 536.40 (442.86) | <0.001 |
| Osteoporosis | | | 0.7 |
| 0 | 262 (29%) | 60 (30%) | 0.005 |
| 1 | 395 (44%) | 68 (34%) | |
| 2 | 236 (26%) | 74 (37%) | |

[1]n / N (%); Mean (SD)

[2]Pearson's Chi-squared test; Wilcoxon rank sum test; Fisher's exact test

**Appendix**

Figure S1: Permutation Importance

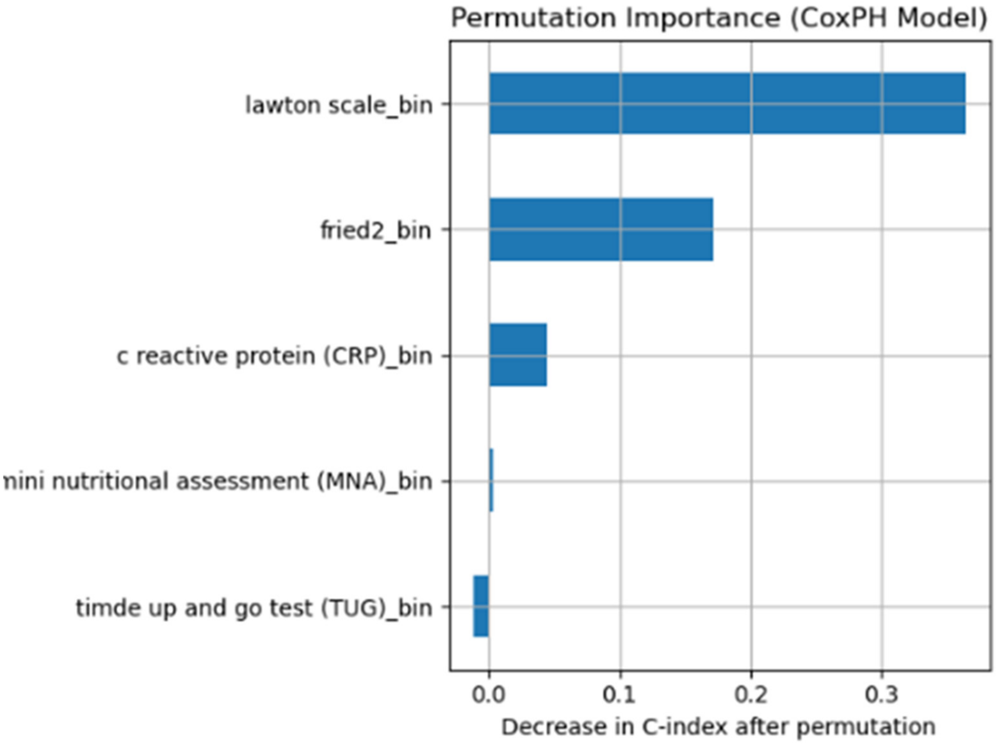


Figure S2: Decision Curve Analysis

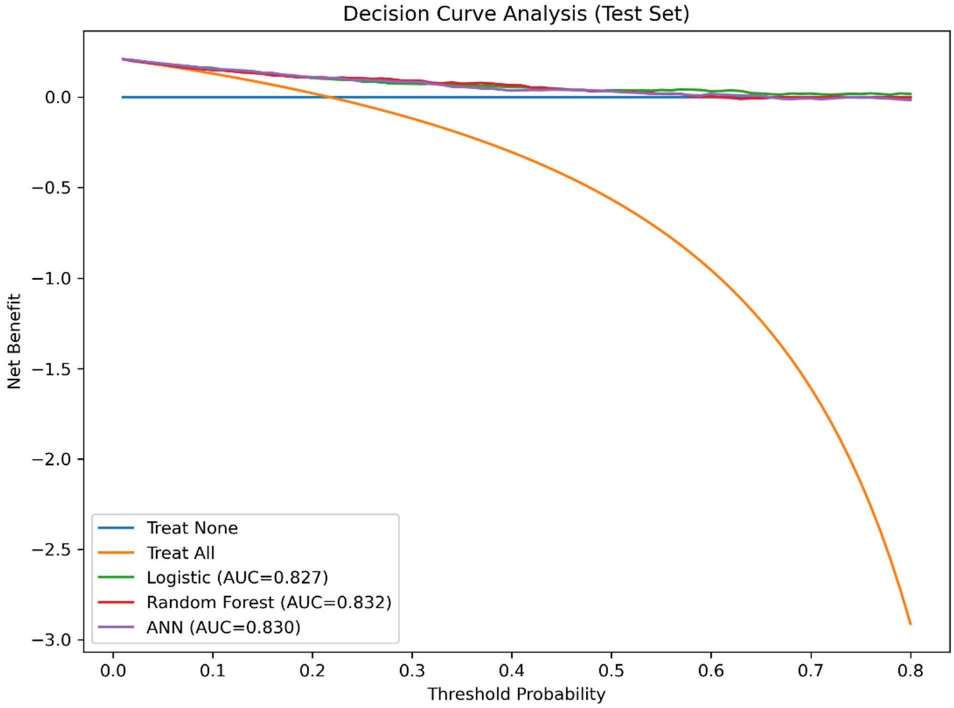

Supplement: Supplementary file 1 [file diagnostics-15-02491-s001.zip › diagnostics-3805513-supplementary.pdf]
